# Structure and Dynamics of dsDNA in Cell-like Environments

**DOI:** 10.3390/e24111587

**Published:** 2022-11-01

**Authors:** Amar Singh, Arghya Maity, Navin Singh

**Affiliations:** Department of Physics, Birla Institute of Technology & Science, Pilani 333031, India

**Keywords:** dsDNA, DNA dynamics, DNA melting, unzipping, crowding, confinement, phase transition, DNA encapsulation, ionic solution

## Abstract

Deoxyribonucleic acid (DNA) is a fundamental biomolecule for correct cellular functioning and regulation of biological processes. DNA’s structure is dynamic and has the ability to adopt a variety of structural conformations in addition to its most widely known double-stranded DNA (dsDNA) helix structure. Stability and structural dynamics of dsDNA play an important role in molecular biology. In vivo, DNA molecules are folded in a tightly confined space, such as a cell chamber or a channel, and are highly dense in solution; their conformational properties are restricted, which affects their thermodynamics and mechanical properties. There are also many technical medical purposes for which DNA is placed in a confined space, such as gene therapy, DNA encapsulation, DNA mapping, etc. Physiological conditions and the nature of confined spaces have a significant influence on the opening or denaturation of DNA base pairs. In this review, we summarize the progress of research on the stability and dynamics of dsDNA in cell-like environments and discuss current challenges and future directions. We include studies on various thermal and mechanical properties of dsDNA in ionic solutions, molecular crowded environments, and confined spaces. By providing a better understanding of melting and unzipping of dsDNA in different environments, this review provides valuable guidelines for predicting DNA thermodynamic quantities and for designing DNA/RNA nanostructures.

## 1. Introduction

The DNA molecule contains genetic instructions used in the development and functioning of all known living organisms. Apart from carrying genetic information and its crucial role in biological processes, it has many applications in drug design, nanotechnology, and nanoelectronics. DNA is often compared to a set of blueprints, similar to a recipe or a code, as it contains the instructions needed to construct other components of cells, such as proteins and ribonucleic acid (RNA) molecules. Proteins and RNA are involved in regulating genetic information through two central and vital processes: *replication* and *transcription* of DNA. In both processes, one thing in common is the opening of the helix, either to serve as a template for daughter DNA (DNA replication) or to decipher the code (DNA transcription). In vitro, this is known as *denaturation* of DNA. In order to understand these important biological processes in depth, one needs to study the opening of the double helix of DNA and the separation of the two strands [1,2].

### 1.1. DNA Double-Helix Opening In Vivo

Replication starts by building a short single-stranded ribonucleic acid (RNA) molecule, which acts as a primer for the duplication process [3,4]. Practically, this means that before each cell division, the DNA sequence must be duplicated. Replication is initiated by an enzyme called DNA-polymerase. It starts by producing a local opening of the base pairs, separating the two strands from each other. The local opening then traverses along the chain like a Y-fork. The bases are matched to synthesize the new partner strands, known as the leading strand and the lagging strand. Many enzymes are involved in the DNA replication fork. Scientists have used a variety of experimental approaches to identify the genes that are crucial in copying DNA [5,6]. It is clear that the genetic content of the DNA molecule is kept protected inside the helix. However, replication requires the strands to be separated [7,8]. Scientists thus started to search for conditions that would disrupt the hydrogen bonds that connect the two strands [9].

The other important phenomenon that involves the opening of DNA base pairs is *transcription*. In this process, the information contained, which forms a gene, is read. During each cell cycle, the DNA sequence is read by an enzyme called RNA-polymerase, and genetic information is transcribed into a messenger RNA (mRNA). This process is called *transcription* [3,4]. The mRNA is then transmitted to another part of the cell and read by a protein-synthesis machine called a ribosome. In order to initiate this process, RNA polymerase has to recognize and connect to a specific site on double-stranded DNA (transcription is controlled by so-called “transcription factors”, which form a pre-initiation complex) that initiates a local opening of the base pairs in hte DNA chain. It is therefore necessary for base-pairing to be only marginally stable so that the helix can be opened and the sequence read. RNA polymerase has the ability to close the bases after copying the sequence. The process is done in a very coordinated manner at a speed of several tens to one hundred base pairs per second [10].

From the above structural and functional discussion of DNA, one finds that the double helix of DNA has several advantages. Here, some of the most relevant aspects are listed:

- Genetic information is coded twice in the two complementary strands. This allows storage of the “information” and the ability to check for errors during replication [9].
- The sugar–phosphate backbone promotes base-paring between complementary strands, which is essential for genetic information storage and retrieval [11].
- The lineal or stacked arrangement of the bases along the longitudinal axis of the DNA allows proteins to directly access the fragment of the sequence.
- Opening (unwinding) and closing of the two DNA strands is reversible. That is how replication and transcription can be carried out without damaging the original molecule.

### 1.2. DNA Double-Helix Opening In Vitro

One can separate the two strands of DNA by increasing the temperature of the solution that contain the DNA molecule. This is known as *thermal melting* or *temperature-induced DNA melting*. In this process, the hydrogen bonds between bases are broken, and the two strands separate from their helix structure. This initiates in the chain with the formation of local *denaturation bubbles* similar to the separation in transcription and replication. Thus, DNA denaturation is seen as a valid approach to understanding the mechanisms of transcription and replication. Despite being distinct from the opening of base pairs that takes place during transcription, comprehending it can nonetheless provide a wealth of helpful information about what occurs throughout this process. Another way to cause separation is by pulling one of the DNA strands while keeping the other strand attached to a glass slide. This is known as *force-induced DNA melting* or *DNA unzipping*.

#### 1.2.1. Thermal Melting of DNA

Soon after the discovery of its structure in 1953, attempts were made to study denaturation. Although the field is in continuous evolution, there are still many open questions regarding DNA; for example: how RNA polymerase searches for and connects to a specific site on double-stranded DNA; how this can be realized in vitro, etc. Moreover, recent developments in experimental techniques have made it possible to manipulate DNA for nanotechnology [12,13,14] and molecular memory [15,16]. Hence, this creates interest in studying DNA’s properties in conditions that are not necessarily physiologically relevant.

**Figure 1:**
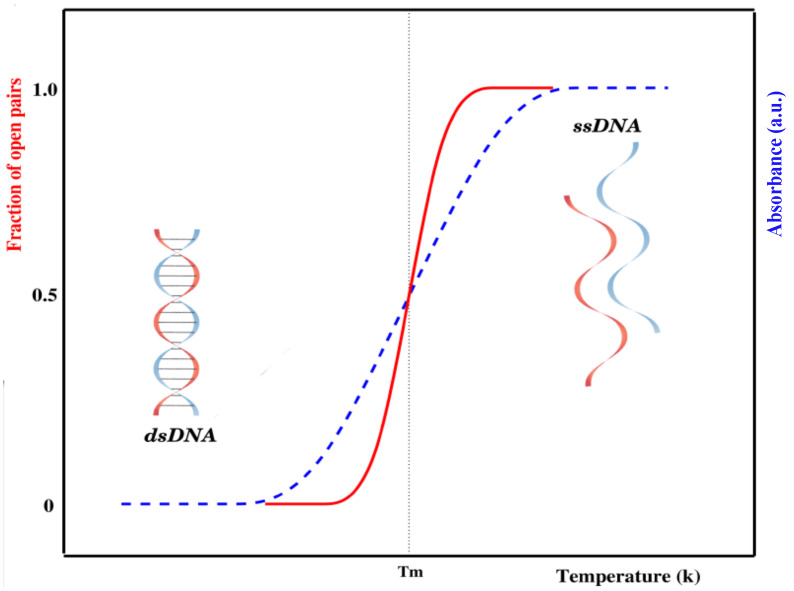
Schematic representation of the melting curve of a short, homogeneous DNA chain either experimentally (generally represented by UV absorbance, shown in blue) or theoretically (generally described by phase transitions with order parameters, i.e., fraction of open pairs, shown in red). The nature of these curves depends on many other factors, e.g., DNA sequence, length, mismatch, etc. [18].

The internal dynamics of DNA has been studied by different experimental methods. The most widely used method for the experimental study of DNA denaturation is UV-absorption spectroscopy [7,17]. An increase in temperature causes a sudden opening of base pairs, which creates bubble(s) in the sequence. Once a bubble is formed, it may grow and hence break other base pairs. This process is similar to the nucleation and propagation of bubbles in crystal structures. The change in stacked bases is consequently accompanied by an abrupt increase in the absorption (intact base pairs in double-stranded DNA absorb less ultraviolet light than bases in a single-stranded chain), as shown in Figure 1.

There is a slight increase in absorbance during the early stages of the transition attributed to a slight increase in the average stacking of base pairs in the double helix. As the temperature is raised, hydrogen-bonded base pairs between complementary strands begin to open. Opening of the remaining base pairs occurs in a highly cooperative manner, causing the strands to completely separate. Once the strands are separated, linear absorbance is again observed if the temperature is further increased. This absorbance corresponds to the increased unstacking of bases in the single strands of DNA. The temperature at which half of the base pairs are open in a DNA chain is known as the melting temperature ($T_{m}$) [18,19]. As a whole, DNA denaturation is an order–disorder transition—the ordered state being given by the intact bases, while the disordered state corresponds to loops formed by broken base pairs [20,21].

#### 1.2.2. Force-Induced Melting of DNA

Force-induced separation of dsDNA strands is closer to the unzipping process occurring in living organisms, where enzymes and/or proteins attach to the molecule and pull it to initiate local opening of the base pairs [22,23]. During replication and transcription, the DNA molecule is severely deformed: the double-helix is untwisted, stretched, and compressed, and the base-pair patterns are locally destroyed. These reversible structural transitions in response to various external conditions are very important for the biological function of DNA, as it is hereditary material. In vitro, at relatively low temperatures where thermal DNA melting does not occur, DNA can be separated by applying an opposite force on the two strands of the DNA molecule [24,25]. This phenomenon is called *force-induced DNA melting* or *DNA unzipping*. In force-induced melting, the force can be applied in a direction either perpendicular or parallel to the helix axis, making force-induced DNA melting directional. When the force is applied perpendicularly, it is commonly referred to as *unzipping* [26,27,28,29]; a parallel force is referred to as a *rupture* [30,31,32,33,34].

In single-molecule force spectroscopy (SMFS) experiments, a DNA molecule is attached to a surface on one end and to a force sensor on the other. The force sensor is usually a trapped micron-sized bead or a cantilever. The displacement of the cantilever measures the force. In a typical unzipping experiment, one of the strands is attached to a glass slide, while the other strand is pulled. There are two main ways to separate the two strands. Either the strand is pulled with a constant velocity (constant-extension ensemble) or with a constant force (constant-force ensemble) [22,35,36]. The microscopic features of unzipping may vary with the choice of ensemble. The thermodynamic limit of these two ensembles are equivalent [37]. In a constant-extension ensemble, the separation between the end base pair of one of the ends in the dsDNA molecule is kept fixed, and the average force needed to keep this separation is measured. In the experimental setup, optical tweezers [38,39] and an atomic force microscope (AFM) [40] essentially control the position of the end bases where the force is applied. In a constant-force ensemble, the force is applied on the end base pair of the strand at a constant rate while keeping base pair on the opposite strand fixed. The distance between two bases in a pair on which force is applied is allowed to fluctuate [41]. This approach is more easily modelled theoretically and may be more closely related to strand separation in cells.

**Figure 2:**
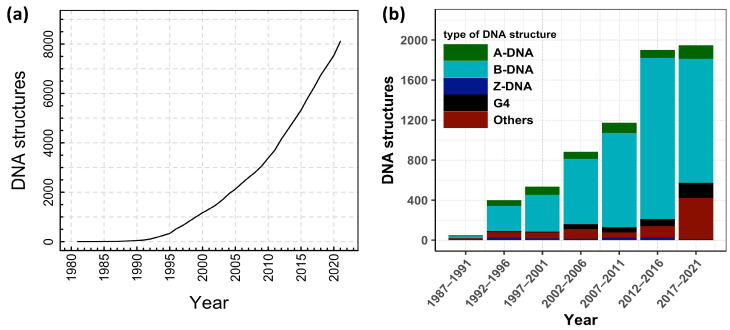
(**a**) Cumulative number of DNA structures from the year 1981 to 2022. (**b**) Fraction of different types of DNA structures released in every five years. Data taken from the Nucleic Acid Database: http://ndbserver.rutgers.edu/, accessed on 27 June 2022.

In order to understand DNA denaturation and DNA unzipping and their various interactions and mechanism in vivo and in vitro, many experiments have been done, and various theories have been proposed. DNA structural studies are also important in DNA nanotechnology in addition to biological fields. There are many possible conformations of DNA, including A-DNA, B-DNA, and Z-DNA, and the conformation that DNA adopts depends on physiological conditions, although the most common form of double helix is B-DNA. Furthermore, a guanine-rich DNA sequence can fold into four-stranded, noncanonical secondary structures called G-quadruplexes. More complex folds of DNA can exist, with extended tertiary structures and enzymatic/catalytic activity. The Protein Data Bank (PDB) and the Nucleic Acid Database (NDB) are two distinct databases from which one can download structures of interest and/or visualize structures using the built-in visualization tools. From NDB, we extracted a number of experimentally verified DNA structures, and we plotted the data in Figure 2.

Figure 2 shows the cumulative number of DNA structures from 1981 to 2022, along with the fraction of different types of DNA structures released every five years. As reflected through the different types of structures in Figure 2b, noncanonical DNA structures have been gaining increasing attention in recent years. These noncanonical structures of nucleic acids, such as G-quadruplex [42], i-motif [43,44], and triplex [45,46,47], might have some functions in vivo, as well as other applications in nanotechnology. However, for this review, we limit ourselves to investigations of DNA double-helix structures only. Further, as DNA research is very vast and is continuously evolutionary, this review is focused on the structure and dynamics of dsDNA either in thermal or force ensembles. We discuss how the presence of cations, other biological crowders, and confined spaces affect the stability of the DNA helical structure under different ensembles.

## 2. Role of Salt Concentration

**Figure 3:**
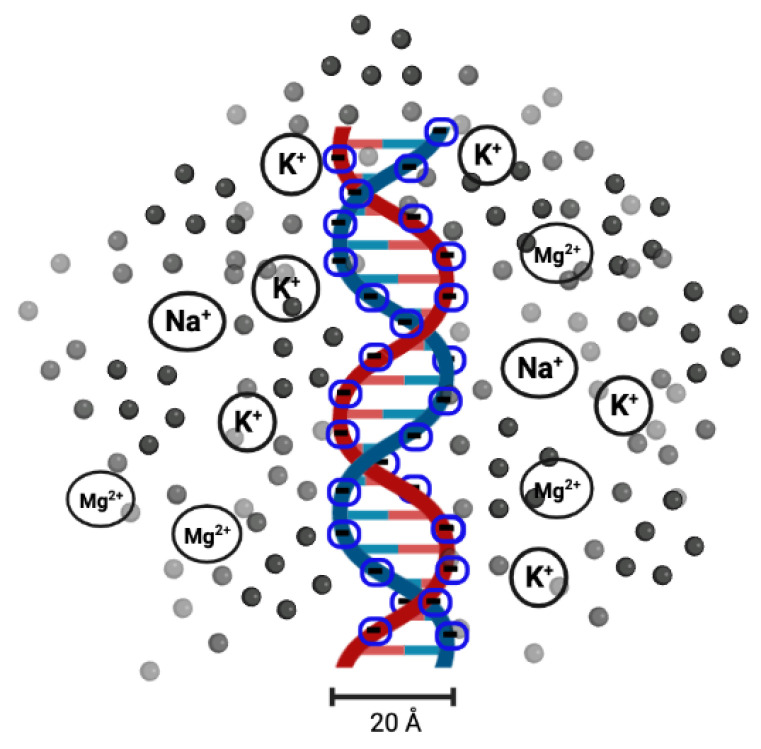
Illustration of the presence of cations (represented by black or gray spheres; not to scale) around the two negatively charged DNA strands. Cations, such as $K^{+}$, $Na^{+}$, $Mg^{2+}$, etc., counteract the repulsion between the two strands as well as the potential distribution of extra cations that interact with one another.

DNA melting has been used as a measure of DNA stability since the 1980s, when melting analysis was first combined with polymerase chain reaction (PCR) [48]. With this technique, a single copy or a few copies of DNA can be amplified to thousands or millions of copies. It is also used to determine the effect of solution conditions. It has been found that the salt (in the form of NaCl or $MgCl_{2}$) concentration of the solution (or ionic solution) influences the melting temperature [49,50,51]. Salt dissolved in the solution releases $Na^{+}$ or $Mg^{2+}$ cations. As the DNA molecules are strong polyelectrolytes, having negatively charged phosphate groups, researchers found that it would be interesting to analyse the role of cations ($Na^{+}$ or $Mg^{2+}$) in the melting transition of dsDNA. The electrostatic free energy change in the transition from double-helix to single-strand is related to the electrostatic repulsion between the phosphate charges on the opposite strands of DNA. The results clearly indicate that the stability of DNA in solutions containing NaCl and $MgCl_{2}$ can be explained by the change in the electrostatic component of the free energy of the double-helix and single-strand conformations. A high concentration of counterions screens the negative charges on the two strands [49,52], as shown in Figure 3.

Several groups of researchers have proposed empirical relations or formulas to predict the experimentally observed salt-dependent melting temperature of a DNA molecule. Frank-Kamenetskii [50] presented a relation to predict the experimental data of Owen et al. related to the salt-dependent melting temperature. J. Santalucia, Jr. compared different nearest-neighbour (NN) parameters used to study salt’s effect on the melting transition in DNA [53]. Later on, such NN parameters were optimized by Barbosa et al. [54] to predict the melting temperature of DNA/RNA hybrids at high and low salt concentrations. These different studies presented different formats of NN thermodynamics. The NN model for nucleic acids assumes that the stability of a given base pair depends on the identity and orientation of neighbouring base pairs. In Barbosa’s studies, a unified set of NN parameters were proposed. In the context of NN and next-NN base-pairs, a recent study investigated how single, double, and triple mismatches influence duplex stability and found that many combinations of multiple mismatches are surprisingly stable [55]. Singh et al. [56] proposed a model Hamiltonian that combines the potential energy of DNA base pairs with a solution term that takes into account the ionic nature of the solution. Their investigations of DNA melting and unzipping are based on the Peyrard–Bishop–Dauxois (PBD) model [57,58], which is a well-known statistical model for describing DNA opening at the base pair level [59,60,61,62]. The model was validated by comparing theoretical results with experimental data and showed good agreement. The sequence heterogeneity, torsional effects, and solvent interaction term were incorporated via Morse potential, and the results demonstrate the differential stability of AT and GC base pairs at low salt concentrations [63,64,65,66]. These proposed methods can be used to calculate melting temperatures (bond strengths) and thermal statistics for DNA molecules of any length in varying ionic solutions. The base-pair opening probabilities in B-DNA were calculated at different salt concentrations by Chen and Prohofsky [67]. They used a model proposed by Prohofsky and co-researchers [68,69]. It was based on self-consistent phonon approximation (SCPA), and real configurations of DNA were considered. H-bonding of bases was represented by Morse potentials as a function of the distance between paired bases, while the other covalent bonds were assumed as harmonic potentials with appropriate parameters.

The presence of other metal ions also affects the structure and dynamics of dsDNA. For a mismatch in a DNA sequence, metal ions are used to bridge the base pairs, which is known as metal-mediated DNA [70]. Zhi-Jie and Shi-Jie used the tightly bound ion theory (TBI) [71] to investigate how metal ions affect the folding stability of B-DNA helices. The basic idea of the model was to separate the tightly bound ions from the diffusive ions in solution. The model explicitly accounts for discrete modes of ion binding and the correlation between tightly bound ions and treats the bulk solvent ions using the Poisson–Boltzmann theory. They quantitatively evaluated the effects of ion concentration, ion size and valence, and helix length on the helix stability. Moreover, they derived practically useful analytical formulas for the thermodynamic parameters as functions of finite helix length, ion type, and ion concentration. They found that helix stability is additive for high ion concentrations and long helices and non-additive for low ion concentrations and short helices [72]. Recently, Silva and Weber [73] used a mesoscopic model and published melting temperatures of sequences containing CC or TT mismatches in the presence of metal ions. These metal-mediated base pairs are becoming increasingly popular in sensing applications and DNA nanotechnology. Another aspect of these metal ions is that they affect the transition between A-DNA and B-DNA. To analyse the effect of ionic valency on the stability of DNA structures, Xue et al. [74] considered different metal ions, including NaCl, $MgCl_{2}$, and $AlCl_{3}$, and carried out molecular dynamic simulations; they concluded that multivalent cations strengthen A-DNA structural stability more than monovalent ions. Electrostatic interactions dominate the interaction between metal ions and DNA. The electropositive metal ions closely coupled to electronegative phosphate groups of DNA chain to shield the electrostatic repulsion between negatively charged DNA strands. Higher metal ionic valencies resulted in stronger interactions between metal ions and DNA. This electrostatic interaction that results from counter-ion dispersal is entirely entropic and not sequence-specific [75]. Despite the varying valency, DNA stability also differs based on the size of metal ions under the same concentration and valency of metal ions. This is due to the fact that larger radii make it difficult to enter the major and minor grooves of DNA [76]. The distributions of ions or molecules (specifically solvent molecules) surrounding helical nucleic acids can be analysed using data derived from molecular dynamics simulations [77,78].

Experimental data relating to the dependence of the melting temperature on *GC* content and concentration of $Na^{+}$ ions in solution were obtained by Owen et al. [79]. Analysis of the experimental findings supports the hypothesis that the change in $T_{m}$ with salt concentration is due to changes in the screened interactions between the negatively charged phosphate groups. For example, the $T_{m}$ values of 92 different DNA sequences were measured over a wide ionic concentration range by R. Owczarzy et al. [80]. They derived a relationship for scaling the $T_{m}$ of DNA duplex oligomers between different ion concentrations. Later on, this group predicted the stability of DNA duplexes in solutions containing magnesium and monovalent cations [81]. The DNA double-helix is also stabilized by stacking interactions between base pairs. P. Yakovchuk et al. studied the contribution of salt-dependent base-pair stacking to DNA duplex stability [82]. Later on, Vuletić et al. [83] explained how DNA conformations change with a decrease in DNA concentration in a very-low-added-salt environment. They also validated the Manning condensation and conductivity theories devised for dilute aqueous polyelectrolytes in the absence of added salt.

The process of thermally induced DNA melting has been studied extensively over the last few decades in the framework of the Poland–Scheraga model (PS model) [84], which was proposed in 1966. In this model, a DNA molecule is considered to be composed of an alternating sequence of bound and denatured states. The bound state is energetically favoured over unbound states, while the loop segment or open states are entropically favoured. Jost and Everaers [85] introduced a local sequence-dependent salt correction of the nearest-neighbour parameters in this model. They compared the predictions of this unified PS model with experimental data. The melting behaviour and probabilities for single base-pair opening of long DNA chains were examined by N. Theodorakopoulos [86] within the framework of the PBD model. In this model, hydrogen bonding between bases is represented by Morse potential, while stacking energies are presented by an anharmonic potential [57,58]. The calculated probabilities for single base-pair opening were consistent with values obtained from experiments.

The first measurements of the entropic elasticity of a single DNA molecule were reported by Smith et al. in 1992. They found that in the low-force regime (<10 pN), the elasticity of dsDNA is entropy-dominated, where the molecule behaves as an ideal polymer with a persistence length of about 50 to 100 base pairs. Soon after, researchers investigated DNA stretching in ionic solutions. Experiments done by Bloomfield’s group predicted that DNA melts during the over-stretching transition [87,88]. In a careful set of experiments, this group showed that changes in solution conditions, i.e., salt concentration, pH, etc., that favour DNA denaturation also reduce the critical force needed to overstretch the molecule [89,90,91]. They found that the persistence length of DNA is reduced in high salt concentrations by electrostatic screening of the repulsive charge along the backbone. The effects of sequence and loop size on hairpin stability have been reported by different groups [92,93,94]. More recently, the effect of monovalent cation size on the thermal stability of DNA hairpins was measured by Earle Stellwagen et al. [95]. The melting temperature decreases with larger cations, as larger cations are less effective in shielding the charged phosphate residues in duplex DNA. This may be due to the fact that the larger cations cannot approach the DNA backbone as closely as smaller cations can.

Mechanical unzipping of DNA using force was first observed by Bockelmann et al. [96,97]. They used λ-phage DNA; in their experiment, one strand of DNA was attached to a glass slide, and the other strand was attached to a micro-needle. The other end of the helix was capped with a hairpin molecule to avoid complete separation. The deflection of the tip was measured on a video image as a function of displacement. They found that typical unzipping forces are in the range of 12–15 pN. The unzipping of dsDNA strongly depends on the ionic strength of the solution. High salt conditions favour the formation and stabilization of the secondary structure of DNA. To understand the conformational behaviour of a giant duplex DNA chain in a mixed solution with various biopolymers with different states of ionization, the structure of the DNA chain was analysed in the presence of polycations, polyanions, and neutral polymers as a model for the cellular environment [98]. A concentrated medium with a neutral polymer induces the discrete folding transition of the DNA, and the addition of small amounts of either the polycation or the polyanion causes structural changes in the folded DNA. The forces exerted by single-stranded binding (SSB) proteins in maintaining the open regions of ssDNA have been measured directly by different groups [99,100].

Huguet et al. [101] experimented with single-molecule force unzipping in a wide variety of conditions, including various salt concentrations, pH values, and temperatures. They determined the 10 unique nearest-neighbour base-pair free energies at different salt concentrations and found that AA/TT stacking energies are strongest, while CC/GG are the weakest. They also measured the unzipping forces for different chain lengths of DNA over a wide range of salt concentrations (0.01 to 1 M NaCl). The unzipping force shows a logarithmic increase with increasing salt concentration of the solution. The elastic properties and secondary structure formation of single-stranded DNA at monovalent and divalent salt conditions were studied by B. Alessandro et al. [102]. For both monovalent and divalent salts, they found that the electrostatic contribution to the persistence length is proportional to the Debye screening length and varies as the inverse of the square root of the cation concentration. The intrinsic persistence length is about 0.7 nm for both types of salts. Divalent cations have been found to be more suitable that monovalent cations for screening electrostatic interactions.

The above-discussed single-molecule experiments have revealed the potential of the techniques for studying DNA unzipping that inspired the development of a theoretical understanding of the problem. Theoretical studies of DNA molecules under mechanical tension treat the transport of the applied stress in DNA and the role of base stacking and structures of stretched DNA in dilute and concentrated solutions. M. Kosikov et al. [103] optimized the configurations of both poly(dA).poly(dT) and poly(dG).poly(dC) homopolymers under high- and low-salt conditions. In order to sample the conformational space as widely as possible, they adopted an implicit representation of the chemical environment with a distance-dependent dielectric constant and atomic charges modified to mimic counterion condensation. They found that the energetic and structural changes in the high-salt regime, which is thought to mimic the natural cellular environment of the double helix, also persist under simulated low-salt conditions. The computed behaviours of poly(dG).poly(dC) and poly(dA).poly(dT) polymers were found to be identical in their findings. This is because they used the simplified nature of the homopolymer model. Podgornik et al. [104] presented a theory that gave the stretching and bending moduli renormalization in the presence of salt. They found that not only the persistence length but also the stretching modulus depend on the salt present in the solution. A. Wynveen and C.N. Likos [105] used molecular dynamics simulations and theoretical calculations to show show that increasing the salt concentration of the solution reduce the range of the forces between the molecular brushes at a given separation between the DNA chains. The theoretical approach was based on a two-dimensional cylindrical cell model. Their findings were consistent with experimental studies of salt-dependent DNA-grafted colloids.

Romano et al. [106] studied the DNA overstretching transition using a recently developed coarse-grained model. They found that overstretching at 23 ${}^{\circ }$C occurs at 74 pN, about 6–7 pN higher than the experimental value at equivalent salt conditions. The 6–7 pN overestimation is due to underestimating the extension at higher forces. Recently, Snodin et al. [107] introduced an extended version of this coarse-grained model to capture the thermodynamic, structural, and mechanical properties of single- and double-stranded DNA. This model can be used for a range of salt concentrations, including those corresponding to physiological conditions (the previous model was parameterized to just one salt concentration). As these unzipping forces are temperature-dependent, scientists investigate the temperature–force phase diagram of DNA in the presence of mono- and multivalent ions [108].

The stability of the DNA helix in an ionic solution is governed by the balance between the attractive and repulsive forces existing in the system. Most of the studies discussed above were performed under low or moderate (0.1–1.0 M) concentrations of salt, and the melting temperature was found to have a logarithmic dependence on the number of cations present in the solution. In a few other experiments [109,110] that were executed at relatively high salt concentrations, some strikingly different behaviour in the DNA molecule was observed. These experiments found that at very high salt concentrations (>1 M), dsDNA molecules were destabilised. Such differential stability of dsDNA was also investigated theoretically using a statistical model by Maity et al. [111]. Through free-energy calculations as a function of temperature and force for a wide range of salt concentrations, they elucidate the cause of the instability of the DNA molecule at higher concentrations. A high salt concentration or a higher number of cations may disturb the balance between positive and negative charge forces, which results in dsDNA destabilization [111]. Such behaviour of DNA stability can also be observed when DNA-conjugated gold nanoparticles are used to synthesize nanostructures [112]. The DNA duplex, bridging two nanoparticles, is significantly destabilized with an increase in the diameter of the nanoparticles, even in the presence of sodium or magnesium ions. This may occur due to partial winding of the DNA duplex onto the nanoparticle, accompanied by disturbance of the secondary DNA structure [112].

## 3. DNA in a Crowded Solution

**Figure 4:**
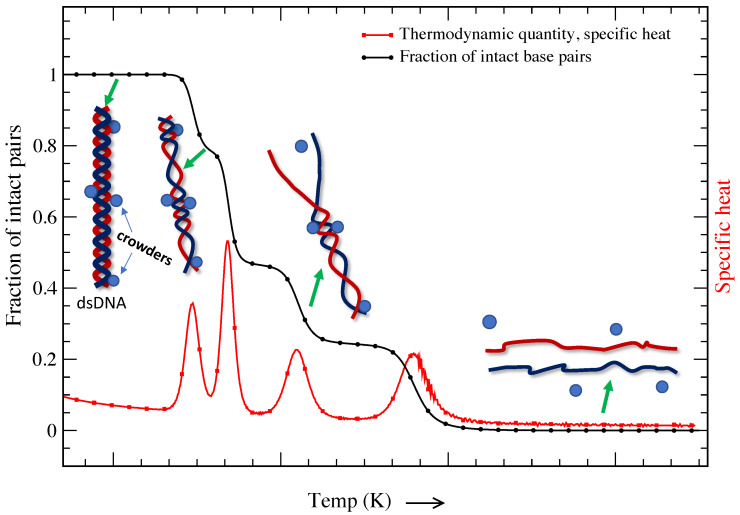
Thermal melting of DNA molecules in the presence of molecular crowders (described in detail in [122]). The change in the fraction of intact pairs with increasing temperature is shown by the black curve, while the change in specific heat is represented by the red curve. To visualize the opening of individual base pairs, corresponding dsDNA or ssDNA chains are displayed and mapped based on the fraction of intact base pairs. The presence of crowders, shown with spheres along the DNA chain, restricts thermal fluctuations, and a further increase in temperature leads to the opening of crowded base pairs.

It is a known fact that the cell consists of a large number of biomolecules, and the volume of the cell is occupied by soluble and non-soluble biomolecules. About 20–40% of the total volume (∼50–400 g/L) of the cell is occupied by nucleic acids, proteins, lipids, saccharides, etc. [113,114,115,116,117]. The presence of such biomolecules is defined as *molecular crowding*, and the volume occupied is known as the *excluded volume*. The presence of crowders in the cell restricts the movement of individual molecules that suppresses thermal fluctuations. It is important to note that DNA melting and other important in vivo cellular processes occur in this crowded environment [118,119,120,121]. Figure 4 is a schematic representation of a DNA molecule’s base pairs crowded by different crowders. The presence of such crowders blocks the propagation of bubbles that are created due to thermal fluctuations; however, further strand fluctuations may displace the crowders from their original locations, opening the previously crowded sites. While DNA transforms from a double-strand configuration to a single-strand configuration smoothly in dilute solution (shown in Figure 1), the transition shows multiple peaks of different heights in a crowded solution. This indicates that the volume excluded by these biomolecules and chemical interactions is critical for determining the structure and stability of DNA molecules. It is interesting to probe how molecular crowding affects the structure and stability of highly ordered DNA structures.

In past years, there has been a growing appreciation of the impact of molecular crowding on DNA duplex stability. The results obtained by different groups are quite interesting and show that the melting temperature of the DNA chain increases about 2–20 ${}^{\circ }$C in a crowded environment. In vitro, poly(ethylene) glycol (EG or PEG) and dextrans are the most commonly used molecules as a cosolute in aqueous solutions to mimic cellular environments. The primary reasons for their use is that they are inert with nucleotides and are available in different molecular weights. Osmolytes are also used to study base-pair stability in crowded solutions [123]. Harve et al. [124] found a variation of 0.5–2.5 K in the increase of melting temperature of 20-oligomer DNA in crowded solution. Nakano et al. reported a decrease in $T_{m}$ with low-molecular-weight PEGs and EGs, while they observed an increase in $T_{m}$ with high-molecular-weight PEGs [125]. Large cosolutes have high steric hindrance, which favours reactions that decrease the net volume; hence, they increase $T_{m}$. Likewise, short cosolutes (ethylene glycol) have lower steric hindrance and decrease the $T_{m}$. In a cellular environment, the surface of a DNA double-helix is coated with layers of water molecules, and any biomolecule that interacts with the DNA must first displace the water sheath [75]. Thus, it is essential to understand what the surrounding water does and how it arranges itself around molecules. However, most theoretical and computational studies are carried out in either a dilute environment or in the absence of water molecules. A recent study demonstrated how water molecules donate or accept hydrogen bonds within prominent parts of the DNA structure, including inside grooves and around the sugar–phosphate backbone [126]. This opens up new possibilities for studying how molecular crowders affect DNA structure and function with the presence of water around DNA. In light of reduced water activity, the role of excluded volume or crowders on the stability the duplex form of DNA can be explained [127,128].

DNA duplex stability studies in crowded ionic environments suggest that the stability of DNA at high polyanion concentrations is significantly increased compared to any other similar ionic strength conditions involving just NaCl or a mixture of NaCl with PEG [110,129]. This results in an additional electrostatic contribution on top of the excluded-volume effect. The decrease in $T_{m}$ at higher ionic concentration is attributed to increased electrostatic repulsion among the DNA phosphates and modification of the electrostatic interactions with counterions. Recently, the effects of loop length on the conformation, thermodynamic stability, and hydration of guanine-rich DNA quadruplexes under molecular crowding were investigated [130]. The effects of molecular crowding with PEP–Na (ploy ethylene sodium phosphate) on the thermodynamics of DNA duplexes, triplexes, and G-quadruplexes were systematically studied [131]. Thermodynamic analysis demonstrated that PEP–Na significantly stabilized the DNA structure. Further, the effect of polymeric solutes on the thermal denaturation behaviour of DNA–gold nanoparticle assemblies was studied by Goodrich et al. [132]. Polymeric solutes can dramatically affect biochemical reactions via molecular crowding. However, both PEG and dextran increase the stability of DNA–gold aggregates, and melting transition temperatures in the presence of PEG are affected more significantly. For a high (∼15%) weight percent of PEG, aggregation was observed even in the absence of complementary oligonucleotides. Recently, the nearest-neighbour parameters were determined by Ghosh et al. [133] to predict the thermodynamics of DNA duplexes in the intracellular environment. Several non-self-complementary and self-complementary DNA sequences of various lengths were taken into consideration, and the researchers established a method to utilize these parameters in any set of crowding conditions. Such methods can be useful for studying biological reactions controlled by specific intracellular crowded conditions. In a recent study by Marimoto et al. [134], the effects of protein-rich environments on the stability of nucleic acid structures were investigated. The experimental results show that the interactions of basic globular proteins or other organic cations with DNA loop structures are different from the interactions with fully matched duplexes. The basic globular proteins stabilize the long internal and bulge loop structures at high concentrations. The molecular environment inside living cells, consisting of various types of proteins, may be one of the factors that can promote the formation of long loops in noncanonical nucleic acid structures.

Theoretical studies on the effect of molecular crowders on the dynamics of DNA are very limited. However, the last few years have witnessed a growing interest in theoretical studies related to DNA dynamics in the presence of crowders. Some of the main areas of focus have been DNA–protein interaction and DNA melting [135,136,137,138]; the effect of an applied force on a polymer in a crowded environment is similar to what is observed in a cell [139,140,141]. All these studies predict that molecular crowding can increase the DNA duplex stability, and the level of stabilization can be different depending on the representation of the denatured state. The other class of studies proves the existence of multi-step transitions in the force–extension curve of polymers. However, the response of a polymer to an applied force remains an elusive problem. Very limited theoretical results investigating the effect of mechanical force on DNA or proteins in a crowded environment are available.

Harve et al., through atomistic molecular dynamics simulations, elucidated that molecular crowding stabilizes hydrogen bonding between complementary nucleotides [124]. Y. Lui et al. [142] developed a thermodynamic model to predict DNA melting in ionic and crowded solutions. Their findings predict an increase of 8 ${}^{\circ }$C in the melting temperatures of dsDNA and DNA—RNA hybrids, which is in good agreement with available simulation and experimental results. In their model, base pairs are represented by two types of charged Lennard Jones spheres. They observed that $T_{m}$ increases in the presence of crowders due to the volume occupied by the crowder molecules, which suppresses the DNA-melting entropy. Their calculations showed that at a given concentration, a larger crowder exhibits greater suppression of entropy, which results in a higher $T_{m}$ of DNA. In this model, water molecules were treated as a background, which implies that the water molecules do not occupy any volume in the system. This may lead to an inaccurate description of the molecular crowder in the solution. As a consequence, a revised model was proposed, and the water molecules were represented explicitly as neutral spherical particles. The interactions between all species, such as dsDNA and ssDNA segments, water, crowders, and ions, were described by Lennard Jones and coulombic potentials [143]. In the presence of crowders, changes in the Gibbs energy, entropy, and enthalpy can be predicted from the model with good agreement with experimental observations.

In a recent work, Brackley et al. [144], using coarse-grained Brownian dynamics (BD) simulations, reported the effect of molecular crowders on the protein–DNA target search process. They studied the effect of the presence of crowders in cytosol and along the DNA molecule. Their findings suggest that a proper account of the crowded cellular environment is crucial for complete understanding of the protein–DNA target search. As a coarse-grained model of DNA, oxDNA captures both the structural and thermodynamic properties of ssDNA and dsDNA. Such coarse-grained models can also be used to study DNA interactions under various crowding conditions. Hong et. al [145] extended the oxDNA model by introducing inert crowding particles. The crowders are represented by spheres interacting with excluded volumes. The same approach for crowder representation was also used earlier by other researchers [122,146]. In this approach, the thermodynamics and kinetics of interactions between crowders and DNA are emphasized based more on the entropic properties of excluded-volume interactions rather than other interactions between crowders and DNA, such as electrostatic attraction. According to these findings, molecular shape and the environment have a peculiar correlation that may be controlled directly by engineering specific confinement devices.

It is believed that the highly crowded environment strongly promotes DNA self-assembly. This leads to extremely condensed and thermodynamically stable DNA aggregates. The G-rich strand of human telomeric DNA can fold into a four-stranded structure called a *G-quadruplex*. Such structural transitions have been observed in vivo and in vitro [121]. The presence of macromolecular crowders (or an increase in excluded volume) decreases the availability of conformational space and conformational distributions, which decreases entropy. The excluded volume can be minimized either by changing the hydrodynamic volume (folding) or by making biomolecular assemblies, thereby stabilizing the ordered structure [147,148]. The effects of molecular crowding on the mechanical stability of protein molecules were studied by Yuan et al. [140]. They found that the mechanical stability of ubiquitin molecules was enhanced by molecular crowding. However, there is a lack of reports on the mechanical stability of DNA molecules under macromolecularly crowded conditions, which prevents interpretation of the biological relevance of these molecule structures. Nevertheless, together with new theoretical studies on biomolecules under mechanical tension, single-molecule force spectroscopy continues to generate new insights into force and its relation to the structure and function of DNA in crowded environment.

All of the studies discussed above indicate the importance of molecular crowding and its impact on designing biosensors, drugs, etc. Despite decades of computational and experimental evidence showing that crowding can significantly affect the kinetics and thermodynamics of biochemical reactions, this topic is still far from complete understanding.

## 4. DNA in Confined Geometry

**Figure 5:**
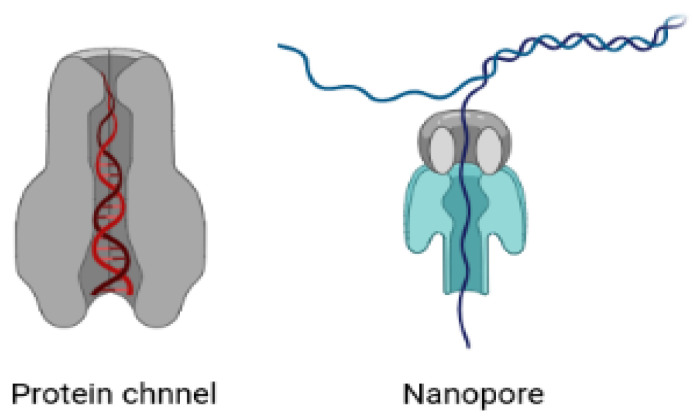
DNA molecule in examples of confined environments: protein channel and nanopore. The thermodynamic properties of DNA molecules highly depend on these confined spaces and on the solvent properties.

In vivo, a DNA molecule is in a highly dense solvent environment and is confined in a limited space, such as a cell chamber or a channel [149,150,151] (see Figure 5). The solvent’s ionic charges make it easy for DNA polymers to slide through the confinement channel [152]. The conformation and movement of DNA molecules are circumscribed by this confinement. Therefore, the limited space and the solvent properties have a significant impact on the thermodynamics of DNA molecules [153]. It is well-recognised that the structural characteristics of biopolymers under confinement have a major impact on biological systems, such as the packing of DNA in eukaryotic chromosomes and viral capsids, among others aspects [154]. We need to understand the denaturation and renaturation mechanisms of DNA in the restricted state in great detail in order to comprehend these features. This research would give us the ability to use DNA for efficient design and control of the molecule’s self-assembly behaviour for a variety of purposes.

The results of experiments have demonstrated that confinement has a significant impact on the entropy of DNA molecules [155]. Zoli et al. [156] used a three-dimensional mesoscopic model to investigate the characteristics of short DNA chains in a confining space. In a fascinating work, Derrington et al. [157] explained how *Mycobacterium smegmatis* porin A (MspA) can be utilized to determine the sequence of DNA. Single-stranded DNA’s individual nucleotides can be identified using MspA, a short and narrow constriction. In a significant paper, Lau et al. [158] demonstrated that a short DNA strand initially contained in a nanotube with a diameter of 4 nm displays dynamics comparable to the unconfined molecule, but the behaviour is significantly altered when the diameter is decreased to 3 nm. When it comes to biological applications and DNA manipulation, carbon nanotubes serve as templates for DNA encapsulation, endocytosis-mediated intracellular penetration, and the transport of biological payloads, among other things [132,154]. Recently, we have witnessed some theoretical and practical research showing the local characteristics of the biopolymers’ translocation in nanochannels [159,160,161,162]. Biopolymer translocation through conical geometry has been the subject of some experimental experiments [163,164]. It is yet unknown which shape provides the best resolution for reading the information along the DNA contour [161,162]. Franceschini et al. [152] demonstrated how the interior channel’s possession of charges aids in the smooth sliding of DNA during the moment of translocation. Their findings suggest that a net internal negative surface charge is crucial for enabling smooth passage of the opposing negatively charged DNA via the connector or beta–clamp temperature when it is used in conjunction with altering the conical geometry’s angle using a statistical model. All of these analyses take into account the confinement’s geometry, which might be either rectangular or spherical [149]. Although there have been recent advances, little is still understood about the trajectory profiles and confinement nature of DNA travelling through these nanopores [160]. The influence of the molecular weight and the confinement dimensions on the diffusion coefficient under the confinement regime has been examined by Gregory T. Morrin et al. [165]. They used single-molecule fluorescence-tracking microscopy to take advantage of convex lens-induced confinement (CLiC) to investigate how the diffusion of small DNA fragments varied as a function of slit height. S. Jonchhe et al. [166] investigated the folding and unfolding transitions of the hairpin DNA duplex inside a DNA origami nanocavity in another recent study utilising a 17-bp DNA duplex in the form of a hairpin stem. They demonstrated that, as compared to the free solution, the mechanical and thermodynamic stabilities of the DNA hairpin inside the nanocage are significantly reduced. According to the study, nanoconfinement unexpectedly reduces the stability of the hairpin DNA duplex.

One of the major obstacles to the effective and efficient use of DNA for various applications such as gene therapy [167], diagnostics [168], and nanorobotics [169], is DNA degradation. DNA degradation occurs via chemical breakdown [167] or sometimes through mechanical forces [170]. As we know, DNA is protected through a physical barrier in the popular gene therapy technique, and in this technique, there are many ways to get better results, such as complexation with polycations [167], charged copolymers of different architecture, and cationic lipids or liposomes [171]. There are also other processes, such as DNA confinement within gels [172,173], polymeric nanocapsules (micelles) [174,175], and microparticles. Hence, DNA encapsulation has been studied through many elegant and versatile approaches [176].

In DNA encapsulation, the abilities to preserve DNA and efficiently release it are inversely related. The objective of good DNA encapsulation is to find an optimal balance between these two issues. Researchers are trying to balance them in numerous ways: one of them is short DNA encapsulated by a spherical inorganic nanoshell with an overall thickness of ∼10 nm [177]. Additionally, carbon nanotubes have demonstrated themselves as a possible candidate for DNA encapsulation [178]. An important field of study is the thermodynamic spontaneity of DNA encapsulation in carbon nanotubes under various circumstances. The threshold diameter of this tube is also a vital issue to investigate, since below the threshold, encapsulation is inhibited [179]. Many sensitive parameters are involved in DNA encapsulation, such as the medium and topology of the carrier, thermodynamic parameters, etc. The stability of DNA molecules during encapsulation is a field of earnest research. Maity et al. [180,181] have studied the thermal stability of double-stranded DNA molecules of different lengths in a confined space. Using the PBD model and molecular dynamics simulations, they evaluated the melting profile of DNA of different lengths in two geometries: conical and cylindrical. Their results show that not only the confinement but also the geometry of the confined space plays a prominent role in the stability and opening of the molecule.

## 5. Translocation of DNA (DNA Passing through Pores)

Traditional *DNA mapping*, which relies on enzyme-based labelling, is very costly and thorough. In contrast, a new concept, *denaturation mapping*, is easy and affordable [182,183]. *Denaturation mapping* primarily relies on the idea that melting in *GC*-rich regions requires more energy than melting in *AT*-rich regions. Reisner et al. determined a DNA sequence by optically mapping denaturing DNA that was contained in a rectangular nanochannel [184]. This method opens up new possibilities for predicting the genome sequence by fusing the experimental approach with a computer simulation built on the Poland–Scheraga model [23], which assesses the sequence-dependent melting probability. In recent research, Maity et al. [185] expanded these ground-breaking studies and made an effort to comprehend how DNA that is completely or partially confined in a nanochannel melts. They studied the melting of homogenous DNA in two distinct geometries: conical and cylindrical. Work pertaining to the translocation of DNA through the nanopores of various geometries was the driving force behind selecting two different geometries.

Numerous biopolymers, such as proteins and nucleic acids, are studied using nanopores [160,163,186,187,188,189,190]. Biotechnological and solid-state nanopores are the two main types used to decipher genomic sequences. The nanopores found in cells are used to control the flow of molecules between the cytoplasm and the nucleoplasm. Their primary job is to control many important processes and to maintain the concentrations of various solutes and the osmotic pressure of the cell. This selective transport of biopolymers is highly regulated via the complex structures of these pores. There has been considerable attention paid by researchers to improve our fundamental understanding of a variety of biological processes [154,182,191,192,193,194]. The knowledge gained opens the way to diverse nanotechnological applications such as DNA sequencing and protein analysis [190,195] and may aid to the development of therapies involving the controlled translocation of biomolecules across the cell membrane (e.g., gene therapy and drug delivery) [196].

In recent years, nanopores have been identified as a promising tool for single-molecule analysis [160,163,197,198]. The structure and the size of nanopores makes them ideal candidates for single-molecule analysis. In typical nanopore experiments, nanopores are placed as the sole fluidic, and voltage is applied across the ionic solution. The current passing through the pore depends upon the size and shape of the nanopore. In one experiment, genetically engineered variants of natural transmembrane protein pores were proposed and found to have the highest sensitivity [162,163,188]. However, solid-state nanopores have the advantage in terms of tunable size and shape. In addition to these studies, a study related to the stability of biopolymers, especially DNA molecules, when they passes through these pores also needs special attention [150,159]. The confined space of the cytoskeleton and cell organelles restricts the movement of DNA molecules and hence alters their stability. There have been considerable experimental attempts to understand the dynamics and stability of DNA molecules that pass through biological or solid state pores [149,155].

J.B. Heng et al. [199] investigated the electromechanical characteristics of DNA on a nanometer-length scale by driving single molecules through artificial nanopores in ultrathin silicon nitride membranes with an electric field. Polyanionic DNA submerged in an electrolyte at the cathode moves toward the anode when an electric field is applied across a synthetic membrane with a nanometer-diameter pore in it, eventually penetrating the membrane through the pore. An exceptionally demanding test of the polymer’s electromechanical characteristics is the translocation of DNA through the pore due to an electric field. They [199] found that at low electric fields, single-stranded DNA can pass through pores that are ≥1.0 nm in diameter, but double-stranded DNA can only pass through pores that are ≥3 nm in diameter. They discovered a double-stranded DNA permeability threshold for pores <3.0 nm in diameter that relies on the electric field and pH.

To investigate the secondary structures of nucleic acids by employing electrical force to unzip duplex areas [200,201], lipid-embedded α-hemolysin channels [202,203,204,205] are utilised as model nanopores. The key drawback that prevents potential biotechnological nanopore applications is the lateral diffusion of these channels in the membrane and the fragility of these lipid bilayers [195,196]. One significant advancement in this area is the use of solid-state nanopores [206,207,208,209,210] for sensing, since they outperform biological nanopores [211,212] in terms of high stability, simple size control, customizable surface features, and the potential for integration into devices and arrays [213,214]. Solid-state nanopores with diameters between those of ssDNA and dsDNA cross-sections (somewhere between 1.5–2 nm) are used to determine the unzipping kinetics of DNA secondary structures by applying a shear force to induce DNA unzipping [215,216,217]. Regarding the direct measurement of single-molecule unzipping kinetics through solid state pores, we still have several gaps to fill. Numerous intriguing findings demonstrate the use of biological pores to unzip hairpin and duplex DNA [215,218]. Recently, Yao Lin et al. studied the unzipping kinetics of dsDNA through sub-2 nm solid-state nanopores [219]. By incorporating the solid-state nanopores into an all-in-one bioanalytical system, DNA unzipping through these nanopores has applications in the realm of clinical and laboratory use of nanopore sensors. We still need further research on solid-state nanopores to show that the patterns that now exist match the entire unzipping process of DNA duplexes.

## 6. Conclusions and Future Directions

In vivo, DNA molecules are folded in a confined space, such as a cell chamber or a channel, and are highly dense in solution; their conformational properties are restricted, which affects their thermodynamics and mechanical properties. In this review article, we attempt to understand the behaviour of DNA molecules in different physiological environments, such as in the presence of crowders, ionic solutions, and confined environments. We first discussed the structure and dynamics of dsDNA in concentrated and ionic solutions. Various experimental and theoretical attempts help us to understand how these ions’ properties, such as charge, size, and concentration, determine the stability and dynamics of DNA molecules. The microscopic properties of the system can be investigated in different ensembles, such as thermal or force ensembles, and many studies have been done so far since the mid-1950s. The stability of the DNA helix in an ionic solution is governed by the balance between the attractive and repulsive forces existing in the system. Electropositive ions closely coupled to electronegative phosphate groups of DNA the chain shield the electrostatic repulsion between negatively charged DNA strands. The findings show a logarithmic dependence of melting temperature and unzipping force based on the salt concentration of the solution. However, a very high salt concentration (>1 M), where the greater number of cations may disturb the balance between positive and negative charge forces, destabilizes double-stranded DNA. The elastic properties and secondary-structure formation of single-stranded DNA at monovalent and divalent salt conditions are also discussed. We try to scrutinize all of these studies in this review for a better understanding of DNA melting and unzipping in ionic environments. Future challenges include quantitative characterization of the energetics of formation of high-order DNA conformations in ionic solutions. For example, DNA duplexes containing G-rich sequences may spontaneously form G4/IM-synaptic complexes. The dynamics of such assemblies with varying salt solutions/concentrations would be interesting to investigate.

We discussed the molecular crowded environment and crowding effects on DNA structure. It is known that a highly crowded environment strongly promotes DNA self-assembly. Many experimental studies have reported the increase of melting temperature of DNA in crowded solutions. DNA duplex stability studied in ionic crowded environments suggests that the stability of DNA at high polyanion concentrations significantly increases compared to any other similar ionic strength conditions involving just NaCl or a mixture of NaCl with PEG. This results in an additional electrostatic contribution on top of the excluded-volume effect. The last few years witnessed a growing interest in theoretical studies related to DNA dynamics in the presence of crowders. Statistical model-based studies and molecular dynamics simulations have both played a role in this. These studies reveal that denaturation and folding in a crowded environment is influenced by both entropic and enthalpic effects. However, the understanding of DNA melting or denaturation in a crowded solution is still far from complete; for example, the response of a polymer to an applied force (constant or time-dependent) remains an elusive problem. At the micrometer level, the mechanical properties of single DNA molecules have been well-characterized and are commonly quantified by a persistence length. However, DNA’s dynamics can only be understood by taking into account its complex mechanical behaviour at different length scales. The scientific community expects more theoretical results investigating the effects of mechanical forces on DNA and RNA in a crowded environment.

In vivo, the DNA molecule is confined in a limited space, and its conformational properties under confinement are of central relevance in living systems. Researchers have shown that the thermodynamic properties of DNA molecules highly depend on the geometry of this space. Experimental findings show that confinement strongly affects the entropy of DNA molecules. Another side of this issue goes with DNA degradation, which is also a major obstacle to the effective and efficient use of DNA. In DNA encapsulation, the ability to preserve the DNA and efficiently release it depends on many sensitive parameters, such as the medium and topology of the carrier, thermodynamic parameters, etc. These studies collectively provide a comprehensive description of dsDNA mechanics in confined spaces by assessing how microscopic base-pair fluctuations impact its thermodynamic properties.

We concentrate on an interconnected issue: DNA passing through different geometrical pores, which is related to many issues such as DNA mapping schemes, gene therapy, drug delivery, etc. Nanopores are frequently utilised to analyse biopolymers, such as proteins and nucleic acids, so there have been considerable experimental attempts to understand the dynamics and stability of DNA molecules passing through biological or solid-state pores. The study is very significant, as the nanopores found in cells control the flow of molecules between the cytoplasm and the nucleoplasm, and research studies show that this selective transport of biopolymers is highly regulated via the complex structures of these pores. The use of solid-state nanopores for sensing is a recent discovery in this field, since they have advantages over biological nanopores in terms of high stability, simple size control, customizable surface features, and the potential for integration into devices and arrays. Improved characterization of DNA nanopores can potentially impact other areas of biology, biophysics, and nanotechnology.

In summary, the structure and dynamics of biomolecules in cell-mimicking environments has gained the attention of researchers. In view of this, we discuss various environment conditions that share a degree of similarity and overlap when it comes to the concepts of excluded volumes. However, physiological relevance and mathematical descriptions differ, and researchers use different approaches to study DNA structural dynamics in crowded and confined spaces. This review offers an improved description of the melting and unzipping of dsDNA in different environments and might serve as a valuable guideline for the future design of DNA and RNA structures. In view of nucleic acid structures being dynamically affected by cellular environments, the mechanism of nucleic acid reactions may need to be redefined. As shown in Figure 2, not only is the cumulative number of experimentally determined DNA structures increasing, but the non-canonical structural conformations of DNA are also increasing. As a result, DNA/RNA molecules will be revealed to be involved in cellular functions, and new technologies will be developed to utilize DNA/RNA in response to environmental factors. 

## Data Availability

Not applicable.

## References

[1] Vologodskii A. (2015). Biophysics of DNA.

[2] Watson J.D., Crick F.H. (1953). Molecular Structure of Nucleic Acids: A Structure for Deoxyribose Nucleic Acid. Nature.

[3] Sinden R.R. (1994). DNA Structure and Function.

[4] Bloomfield V.A., Crothers D.M., Tinoco I., Hearst J.E., Wemmer D.E., Killman P.A., Turner D.H. (2000). Nucleic Acids: Structures, Properties, and Functions.

[5] Omoto C. (2004). Genes and DNA: A Beginner’s Guide to Genetics and Its Applications.

[6] Anders M. (2018). DNA, Genes, and Chromosomes.

[7] Calladine C.R., Drew H., Luisi B., Travers A. (2004). Understanding DNA, Third Edition: The Molecule and How it Works.

[8] Carlberg C., Molnar F. (2014). Mechanisms of Gene Regulation.

[9] Watson J., Myers R., Myers U., Caudy A., Witkowski J. (2007). Recombinant DNA: Genes and Genomes: A Short Course.

[10] Gaeta G. (1999). Results and Limitations of the Soliton Theory of DNA Transcription. J. Biol. Phys..

[11] Poltev V., Anisimov V.M., Danilov V.I., Garcia D., Sanchez C., Deriabina A., Gonzalez E., Rivas F., Polteva N. (2014). The role of molecular structure of sugar-phosphate backbone and nucleic acid bases in the formation of single-stranded and double-stranded DNA structures. Biopolymers.

[12] Komiya K., Yamamura M., Rose J.A. (2010). Quantitative design and experimental validation for a single-molecule DNA nanodevice transformable among three structural states. Nucleic Acids Res..

[13] Chen Y., Lee S.H., Mao C. (2004). A DNA Nanomachine Based on a Duplex–Triplex Transition. Angew. Chem. Int. Ed..

[14] Takinoue M., Suyama A. (2006). Hairpin-DNA memory Using Molecular Addressing. Small.

[15] Takinoue M., Suyama A. (2004). Molecular reactions for a molecular memory based on hairpin DNA. Chem-Bio Inf. J..

[16] Liu D., Balasubramanian S. (2003). A Proton-Fuelled DNA Nanomachine. Angew. Chem. Int. Ed..

[17] Cantor C.R., Schimmel P.R. (1980). Biophysical Chemistry: Part III: The Behavior of Biological Macromolecules (Their Biophysical Chemistry).

[18] Wartell R.M., Benight A.S. (1985). Thermal denaturation of {DNA} molecules: A comparison of theory with experiment. Phys. Rep..

[19] Nelson P. (2003). Biological Physics: Energy, Information, Life.

[20] Zimm B.H. (1960). Theory of Melting of the Helical Form in Double Chains of the DNA. J. Chem. Phys..

[21] Poland D., Scheraga H.A. (1970). Theory of Helix-Coil Transitions in Biopolymers; Statistical Mechanical Theory of Order-Disorder Transitions in Biological Macromolecules.

[22] Kumar S., Li M.S. (2010). Biomolecules under mechanical force. Phys. Rep..

[23] Frank-Kamenetskii M.D., Prakash S. (2014). Fluctuations in the {DNA} double helix: A critical review. Phys. Life Rev..

[24] Essevaz-Roulet B., Bockelmann U., Heslot F. (1997). Mechanical separation of the complementary strands of DNA. Proc. Natl. Acad. Sci. USA.

[25] Sebastian K.L. (2000). Pulling a polymer out of a potential well and the mechanical unzipping of DNA. Phys. Rev. E.

[26] Lubensky D.K., Nelson D.R. (2000). Pulling Pinned Polymers and Unzipping DNA. Phys. Rev. Lett..

[27] Bhattacharjee S.M. (2000). Unzipping DNAs: Towards the first step of replication. J. Phys. A Math. Gen..

[28] Kapri R., Bhattacharjee S.M., Seno F. (2004). Complete Phase Diagram of DNA Unzipping: Eye, *Y* Fork, and Triple Point. Phys. Rev. Lett..

[29] Srivastava S., Singh N. (2011). The probability analysis of opening of DNA. J. Chem. Phys..

[30] Chakrabarti B., Nelson D.R. (2009). Shear Unzipping of DNA. J. Phys. Chem. B.

[31] Santosh M., Maiti P.K. (2009). Force induced DNA melting. J. Phys. Condens. Matter.

[32] Prakash S., Singh Y. (2011). Shear unzipping of double-stranded DNA. Phys. Rev. E.

[33] Mishra R.K., Modi T., Giri D., Kumar S. (2015). On the rupture of DNA molecule. J. Chem. Phys..

[34] Tee S.R., Wang Z. (2018). How Well Can DNA Rupture DNA? Shearing and Unzipping Forces inside DNA Nanostructures. ACS Omega.

[35] Singh A., Singh N. (2015). Pulling DNA: The Effect of Chain Length on the Mechanical Stability of DNA Chain. Macromol. Symp..

[36] Singh A., Mittal B., Singh N. (2013). Force induced unzipping of dsDNA: The solvent effect. Phys. Express.

[37] Singh N., Singh Y. (2005). Statistical theory of force-induced unzipping of DNA. Eur. Phys. J. E.

[38] Simmons R., Finer J., Chu S., Spudich J. (1996). Quantitative measurements of force and displacement using an optical trap. Biophys. J..

[39] Rico-Pasto M., Ritort F. (2022). Temperature-dependent elastic properties of DNA. Biophys. Rep..

[40] Binnig G., Quate C.F., Gerber C. (1986). Atomic Force Microscope. Phys. Rev. Lett..

[41] Weeks J., Lucks J., Kafri Y., Danilowicz C., Nelson D., Prentiss M. (2005). Pause Point Spectra in {DNA} Constant-Force Unzipping. Biophys. J..

[42] Nishio M., Tsukakoshi K., Ikebukuro K. (2021). G-quadruplex: Flexible conformational changes by cations, pH, crowding and its applications to biosensing. Biosens. Bioelectron..

[43] Gao B., Hou X.M. (2021). Opposite Effects of Potassium Ions on the Thermal Stability of i-Motif DNA in Different Buffer Systems. ACS Omega.

[44] Brown S.L., Kendrick S. (2021). The i-Motif as a Molecular Target: More Than a Complementary DNA Secondary Structure. Pharmaceuticals.

[45] Lin P.Y., Chi R., Wu Y.L., Ho J.a.A. (2022). Applications of triplex DNA nanostructures in sensor development. Anal. Bioanal. Chem..

[46] Bhattacharjee S.M., Foster D.P. (2021). Efimov-DNA phase diagram: Three stranded DNA on a cubic lattice. J. Chem. Phys..

[47] Maji J., Bhattacharjee S.M., Seno F., Trovato A. (2014). Melting behavior and different bound states in three-stranded DNA models. Phys. Rev. E.

[48] Wittwer C.T. (2009). High-resolution DNA melting analysis: Advancements and limitations. Hum. Mutat..

[49] Schildkraut C., Lifson S. (1965). Dependence of the melting temperature of DNA on salt concentration. Biopolymers.

[50] Frank-Kamenetskii M.D. (1971). Simplification of the empirical relationship between melting temperature of DNA, its GC content and concentration of sodium ions in solution. Biopolymers.

[51] Weber G. (2006). Sharp DNA denaturation due to solvent interaction. EPL Europhys. Lett..

[52] Manning G.S. (2001). Counterion Condensation on a Helical Charge Lattice. Macromolecules.

[53] SantaLucia J. (1998). A unified view of polymer, dumbbell, and oligonucleotide DNA nearest-neighbor thermodynamics. Proc. Natl. Acad. Sci. USA.

[54] Basílio Barbosa V., de Oliveira Martins E., Weber G. (2019). Nearest-neighbour parameters optimized for melting temperature prediction of DNA/RNA hybrids at high and low salt concentrations. Biophys. Chem..

[55] Oliveira L.M., Long A.S., Brown T., Fox K.R., Weber G. (2020). Melting temperature measurement and mesoscopic evaluation of single, double and triple DNA mismatches. Chem. Sci..

[56] Singh A., Singh N. (2013). Phase diagram of mechanically stretched DNA: The salt effect. Physics A.

[57] Dauxois T., Peyrard M., Bishop A.R. (1993). Entropy-driven DNA denaturation. Phys. Rev. E.

[58] Peyrard M., Bishop A.R. (1989). Statistical mechanics of a nonlinear model for DNA denaturation. Phys. Rev. Lett..

[59] Rodrigues Leal M., Weber G. (2020). Sharp DNA denaturation in a helicoidal mesoscopic model. Chem. Phys. Lett..

[60] Zoli M. (2021). Base pair fluctuations in helical models for nucleic acids. J. Chem. Phys..

[61] Valle-Orero J., Wildes A.R., Theodorakopoulos N., Cuesta-López S., Garden J.L., Danilkin S., Peyrard M. (2014). Thermal denaturation of A-DNA. New J. Phys..

[62] Singh A., Singh N. (2015). Pulling short DNA molecules having defects on different locations. Phys. Rev. E.

[63] Zoli M. (2011). Thermodynamics of twisted DNA with solvent interaction. J. Chem. Phys..

[64] Singh A., Singh N. (2015). Effect of salt concentration on the stability of heterogeneous {DNA}. Physics A.

[65] Ferreira I., Amarante T.D., Weber G. (2015). DNA terminal base pairs have weaker hydrogen bonds especially for AT under low salt concentration. J. Chem. Phys..

[66] Macedo D., Guedes I., Albuquerque E. (2014). Thermal properties of a DNA denaturation with solvent interaction. Physics A.

[67] Chen Y., Prohofsky E. (1993). Salt dependent premelting base pair opening probabilities of B and Z {DNA} Poly [d(G-C)] and significance for the B-Z transition. Biophys. J..

[68] Gao Y., Prohofsky E.W. (1984). A modified self-consistent phonon theory of hydrogen bond melting. J. Chem. Phys..

[69] Gao Y., Devi-Prasad K.V., Prohofsky E.W. (1984). A self-consistent microscopic theory of hydrogen bond melting with application to poly(dG).poly(dC). J. Chem. Phys..

[70] Jash B., Müller J. (2017). Metal-Mediated Base Pairs: From Characterization to Application. Chem.—A Eur. J..

[71] Tan Z.J., Chen S.J. (2005). Electrostatic correlations and fluctuations for ion binding to a finite length polyelectrolyte. J. Chem. Phys..

[72] Tan Z.J., Chen S.J. (2006). Nucleic Acid Helix Stability: Effects of Salt Concentration, Cation Valence and Size, and Chain Length. Biophys. J..

[73] Silva L.G., Weber G. (2022). Mesoscopic model confirms strong base pair metal mediated bonding for T–Hg2+–T and weaker for C–Ag+–C. Chem. Phys. Lett..

[74] Xue J., Wang P., Li X., Tan R., Zong W. (2022). Transformation characteristics of A-DNA in salt solution revealed through molecular dynamics simulations. Biophys. Chem..

[75] Privalov P.L., Crane-Robinson C. (2018). Forces maintaining the DNA double helix and its complexes with transcription factors. Prog. Biophys. Mol. Biol..

[76] Xue J., Li X., Tan R., Zong W. (2022). Molecular dynamics simulations of A-DNA in bivalent metal ions salt solution. Chin. Phys. B.

[77] Lavery R., Maddocks J.H., Pasi M., Zakrzewska K. (2014). Analyzing ion distributions around DNA. Nucleic Acids Res..

[78] Pasi M., Maddocks J.H., Lavery R. (2015). Analyzing ion distributions around DNA: Sequence-dependence of potassium ion distributions from microsecond molecular dynamics. Nucleic Acids Res..

[79] Owen R.J., Hill L.R., Lapage S.P. (1969). Determination of DNA base compositions from melting profiles in dilute buffers. Biopolymers.

[80] Owczarzy R., You Y., Moreira B.G., Manthey J.A., Huang L., Behlke M.A., Walder J.A. (2004). Effects of Sodium Ions on DNA Duplex Oligomers: Improved Predictions of Melting Temperatures. Biochemistry.

[81] Owczarzy R., Moreira B.G., You Y., Behlke M.A., Walder J.A. (2008). Predicting Stability of DNA Duplexes in Solutions Containing Magnesium and Monovalent Cations. Biochemistry.

[82] Yakovchuk P., Protozanova E., Frank-Kamenetskii M.D. (2006). Base-stacking and base-pairing contributions into thermal stability of the DNA double helix. Nucleic Acids Res..

[83] Vuletić T., Babić S.D., Grgičin D., Aumiler D., Rädler J., Livolant F., Tomić S. (2011). Manning free counterion fraction for a rodlike polyion: Aqueous solutions of short DNA fragments in presence of very low added salt. Phys. Rev. E.

[84] Poland D., Scheraga H.A. (1966). Occurrence of a Phase Transition in Nucleic Acid Models. J. Chem. Phys..

[85] Jost D., Everaers R. (2009). A Unified Poland-Scheraga Model of Oligo- and Polynucleotide DNA Melting: Salt Effects and Predictive Power. Biophys. J..

[86] Theodorakopoulos N. (2010). Melting of genomic DNA: Predictive modeling by nonlinear lattice dynamics. Phys. Rev. E.

[87] Rouzina I., Bloomfield V.A. (2001). Force-Induced Melting of the {DNA} Double Helix 1. Thermodynamic Analysis. Biophys. J..

[88] Rouzina I., Bloomfield V.A. (2001). Force-Induced Melting of the {DNA} Double Helix. 2. Effect of Solution Conditions. Biophys. J..

[89] Wenner J.R., Williams M.C., Rouzina I., Bloomfield V.A. (2002). Salt Dependence of the Elasticity and Overstretching Transition of Single {DNA} Molecules. Biophys. J..

[90] Shokri L., McCauley M.J., Rouzina I., Williams M.C. (2008). {DNA} Overstretching in the Presence of Glyoxal: Structural Evidence of Force-Induced {DNA} Melting. Biophys. J..

[91] Williams M.C., Wenner J.R., Rouzina I., Bloomfield V.A. (2001). Effect of pH on the Overstretching Transition of Double-Stranded DNA: Evidence of Force-Induced {DNA} Melting. Biophys. J..

[92] Vallone P.M., Paner T.M., Hilario J., Lane M.J., Faldasz B.D., Benight A.S. (1999). Melting studies of short DNA hairpins: Influence of loop sequence and adjoining base pair identity on hairpin thermodynamic stability. Biopolymers.

[93] Blommers M.J.J., Walters J.A.L.I., Haasnoot C.A.G., Aelen J.M.A., Van der Marel G.A., Van Boom J.H., Hilbers C.W. (1989). Effects of base sequence on the loop folding in DNA hairpins. Biochemistry.

[94] Antao V.P., Lai S.Y., Tinoco I. (1991). A thermodynamic study of unusually stable RNA and DNA hairpins. Nucleic Acids Res..

[95] Stellwagen E., Muse J.M., Stellwagen N.C. (2011). Monovalent Cation Size and DNA Conformational Stability. Biochemistry.

[96] Bockelmann U., Essevaz-Roulet B., Heslot F. (1997). Molecular Stick-Slip Motion Revealed by Opening DNA with Piconewton Forces. Phys. Rev. Lett..

[97] Bockelmann U., Essevaz-Roulet B., Heslot F. (1998). DNA strand separation studied by single molecule force measurements. Phys. Rev. E.

[98] Kidoaki S., Yoshikawa K. (1999). Folding and unfolding of a giant duplex-DNA in a mixed solution with polycations, polyanions and crowding neutral polymers. Biophys. Chem..

[99] Ritort F. (2006). Single-molecule experiments in biological physics: Methods and applications. J. Phys. Condens. Matter.

[100] Hatch K., Danilowicz C., Coljee V., Prentiss M. (2008). Measurement of the salt-dependent stabilization of partially open DNA by Escherichia coli SSB protein. Nucleic Acids Res..

[101] Huguet J.M., Bizarro C.V., Forns N., Smith S.B., Bustamante C., Ritort F. (2010). Single-molecule derivation of salt dependent base-pair free energies in DNA. Proc. Natl. Acad. Sci. USA.

[102] Bosco A., Camunas-Soler J., Ritort F. (2014). Elastic properties and secondary structure formation of single-stranded DNA at monovalent and divalent salt conditions. Nucleic Acids Res..

[103] Kosikov K.M., Gorin A.A., Zhurkin V.B., Olson W.K. (1999). {DNA} stretching and compression: Large-scale simulations of double helical structures1. J. Mol. Biol..

[104] Podgornik R., Hansen P.L., Parsegian V.A. (2000). Elastic moduli renormalization in self-interacting stretchable polyelectrolytes. J. Chem. Phys..

[105] Wynveen A., Likos C.N. (2010). Interactions between planar polyelectrolyte brushes: Effects of stiffness and salt. Soft Matter.

[106] Romano F., Chakraborty D., Doye J.P.K., Ouldridge T.E., Louis A.A. (2013). Coarse-grained simulations of DNA overstretching. J. Chem. Phys..

[107] Snodin B.E.K., Randisi F., Mosayebi M., Šulc P., Schreck J.S., Romano F., Ouldridge T.E., Tsukanov R., Nir E., Louis A.A. (2015). Introducing improved structural properties and salt dependence into a coarse-grained model of DNA. J. Chem. Phys..

[108] Amnuanpol S. (2017). Ionic effects on the temperature–force phase diagram of DNA. J. Biol. Phys..

[109] Sebastian T., Sarkar M., Ratilainen T., Wittung P., Nielsen P.E., Norden B., Graslund A. (1996). Ionic Effects on the Stability and Conformation of Peptide Nucleic Acid Complexes. J. Am. Chem. Soc..

[110] Khimji I., Shin J., Liu J. (2013). DNA duplex stabilization in crowded polyanion solutions. Chem. Commun..

[111] Maity A., Singh A., Singh N. (2017). Differential stability of DNA based on salt concentration. Eur. Biophys. J..

[112] Sokolov P.A., Ramazanov R.R., Rolich V.I., Popova M.A., Shalygin V.E., Kasyanenko N.A. (2020). Stabilization of DNA by sodium and magnesium ions during the synthesis of DNA-bridged gold nanoparticles. Nanotechnology.

[113] Fulton A.B. (1982). How crowded is the cytoplasm?. Cell.

[114] Zimmerman S.B., Minton A.P. (1993). Macromolecular Crowding: Biochemical, Biophysical, and Physiological Consequences. Annu. Rev. Biophys. Biomol. Struct..

[115] Miyoshi D., Sugimoto N. (2008). Molecular crowding effects on structure and stability of DNA. Biochimie.

[116] Hancock R. (2014). Structures and functions in the crowded nucleus: New biophysical insights. Front. Phys..

[117] Skóra T., Vaghefikia F., Fitter J., Kondrat S. (2020). Macromolecular Crowding: How Shape and Interactions Affect Diffusion. J. Phys. Chem. B.

[118] Ellis R. (2001). Macromolecular crowding: Obvious but underappreciated. Trends Biochem. Sci..

[119] Ellis R. (2001). Macromolecular crowding: An important but neglected aspect of the intracellular environment. Curr. Opin. Struct. Biol..

[120] Pincus D.L., Thirumalai D. (2013). Force-Induced Unzipping Transitions in an Athermal Crowded Environment. J. Phys. Chem. B.

[121] Nakano S., Miyoshi D., Sugimoto N. (2014). Effects of Molecular Crowding on the Structures, Interactions, and Functions of Nucleic Acids. Chem. Rev..

[122] Singh A., Singh N. (2017). DNA melting in the presence of molecular crowders. Phys. Chem. Chem. Phys..

[123] Spink C.H., Garbett N., Chaires J.B. (2007). Enthalpies of {DNA} melting in the presence of osmolytes. Biophys. Chem..

[124] Harve K.S., Lareu R., Rajagopalan R., Raghunath M. (2010). Understanding how the crowded interior of cells stabilizes DNA/DNA and DNA/RNA hybrids–in silico predictions and in vitro evidence. Nucleic Acids Res..

[125] Nakano S., Karimata H., Ohmichi T., Kawakami J., Sugimoto N. (2004). The Effect of Molecular Crowding with Nucleotide Length and Cosolute Structure on DNA Duplex Stability. J. Am. Chem. Soc..

[126] Harp J.M., Coates L., Sullivan B., Egli M. (2021). Water structure around a left-handed Z-DNA fragment analyzed by cryo neutron crystallography. Nucleic Acids Res..

[127] Spink C.H., Chaires J.B. (1999). Effects of Hydration, Ion Release, and Excluded Volume on the Melting of Triplex and Duplex DNA. Biochemistry.

[128] Spink C.H., Chaires J.B. (1995). Selective Stabilization of Triplex DNA by Poly(ethylene glycols). J. Am. Chem. Soc..

[129] Karimata H., Nakano S., Sugimoto N. (2007). Effects of Polyethylene Glycol on DNA Duplex Stability at Different NaCl Concentrations. Bull. Chem. Soc. Jpn..

[130] Fujimoto T., Nakano S., Sugimoto N., Miyoshi D. (2013). Thermodynamics-Hydration Relationships within Loops That Affect G-Quadruplexes under Molecular Crowding Conditions. J. Phys. Chem. B.

[131] Moriyama R., Iwasaki Y., Miyoshi D. (2015). Stabilization of DNA Structures with Poly(ethylene sodium phosphate). J. Phys. Chem. B.

[132] Goodrich G.P., Helfrich M.R., Overberg J.J., Keating C.D. (2004). Effect of Macromolecular Crowding on DNA:Au Nanoparticle Bioconjugate Assembly. Langmuir.

[133] Ghosh S., Takahashi S., Ohyama T., Endoh T., Tateishi-Karimata H., Sugimoto N. (2020). Nearest-neighbor parameters for predicting DNA duplex stability in diverse molecular crowding conditions. Proc. Natl. Acad. Sci. USA.

[134] Morimoto R., Horita M., Yamaguchi D., Nakai H., Nakano S.I. (2022). Evaluation of weak interactions of proteins and organic cations with DNA duplex structures. Biophys. J..

[135] Qin S., Zhou H.X. (2010). Generalized fundamental measure theory for atomistic modeling of macromolecular crowding. Phys. Rev. E.

[136] Mittal J., Best R.B. (2010). Dependence of Protein Folding Stability and Dynamics on the Density and Composition of Macromolecular Crowders. Biophys. J..

[137] Tsao D., Dokholyan N.V. (2010). Macromolecular crowding induces polypeptide compaction and decreases folding cooperativity. Phys. Chem. Chem. Phys..

[138] Batra J., Xu K., Zhou H.X. (2009). Nonadditive effects of mixed crowding on protein stability. Proteins Struct. Funct. Bioinform..

[139] Singh A.R., Giri D., Kumar S. (2009). Effects of molecular crowding on stretching of polymers in poor solvent. Phys. Rev. E.

[140] Yuan J.M., Chyan C.L., Zhou H.X., Chung T.Y., Peng H., Ping G., Yang G. (2008). The effects of macromolecular crowding on the mechanical stability of protein molecules. Protein Sci..

[141] Kumar S., Mishra G. (2011). Stretching single stranded DNA. Soft Matter.

[142] Liu Y., Kermanpour F., Liu H.L., Hu Y., Shang Y.Z., Sandler S.I., Jiang J.W. (2010). Molecular Thermodynamic Model for DNA Melting in Ionic and Crowded Solutions. J. Phys. Chem. B.

[143] Liu Y., Shang Y., Liu H., Hu Y., Jiang J. (2012). Crowding effect on DNA melting: A molecular thermodynamic model with explicit solvent. Phys. Chem. Chem. Phys..

[144] Brackley C.A., Cates M.E., Marenduzzo D. (2013). Intracellular Facilitated Diffusion: Searchers, Crowders, and Blockers. Phys. Rev. Lett..

[145] Hong F., Schreck J.S., Šulc P. (2020). Understanding DNA interactions in crowded environments with a coarse-grained model. Nucleic Acids Res..

[146] Zoli M. (2019). DNA size in confined environments. Phys. Chem. Chem. Phys..

[147] Kuznetsova I.M., Turoverov K.K., Uversky V.N. (2014). What Macromolecular Crowding Can Do to a Protein. Int. J. Mol. Sci..

[148] Minton A.P. (2000). Effect of a Concentrated “Inert” Macromolecular Cosolute on the Stability of a Globular Protein with Respect to Denaturation by Heat and by Chaotropes: A Statistical-Thermodynamic Model. Biophys. J..

[149] Li H., Wang Z., Li N., He X., Liang H. (2014). Denaturation and renaturation behaviors of short DNA in a confined space. J. Chem. Phys..

[150] Kumar S., Kumar S., Giri D., Nath S. (2017). Statistical mechanics of a polymer chain attached to the interface of a cone-shaped channel. EPL Europhys. Lett..

[151] Turner S.W.P., Cabodi M., Craighead H.G. (2002). Confinement-Induced Entropic Recoil of Single DNA Molecules in a Nanofluidic Structure. Phys. Rev. Lett..

[152] Franceschini L., Brouns T., Willems K., Carlon E., Maglia G. (2016). DNA Translocation through Nanopores at Physiological Ionic Strengths Requires Precise Nanoscale Engineering. ACS Nano.

[153] Akabayov B., Akabayov S., Lee S., Wagner G., Richardson C. (2013). Impact of macromolecular crowding on DNA replication. Nat. Commun..

[154] Kumar H., Lansac Y., Glaser M.A., Maiti P.K. (2011). Biopolymers in nanopores: Challenges and opportunities. Soft Matter.

[155] Nakano S., Yamaguchi D., Sugimoto N. (2018). Thermal stability and conformation of DNA and proteins under the confined condition in the matrix of hydrogels. Mol. Biol. Rep..

[156] Zoli M. (2020). Stretching DNA in hard-wall potential channels. EPL Europhys. Lett..

[157] Derrington I.M., Butler T.Z., Collins M.D., Manrao E., Pavlenok M., Niederweis M., Gundlach J.H. (2010). Nanopore DNA sequencing with MspA. Proc. Natl. Acad. Sci. USA.

[158] Lau E., Lightstone F., Colvin M. (2005). Dynamics of DNA Encapsulated in a Hydrophobic Nanotube. Chem. Phys. Lett..

[159] Mogurampelly S., Maiti P.K. (2013). Translocation and encapsulation of siRNA inside carbon nanotubes. J. Chem. Phys..

[160] Wanunu M. (2012). Nanopores: A journey towards DNA sequencing. Phys. Life Rev..

[161] Singer A., Rapireddy S., Ly D.H., Meller A. (2012). Electronic Barcoding of a Viral Gene at the Single-Molecule Level. Nano Lett..

[162] Bell N.A., Keyser U.F. (2016). Digitally encoded DNA nanostructures for multiplexed, single-molecule protein sensing with nanopores. Nat. Nanotechnol..

[163] Bell N., Chen K., Ghosal S., Ricci M., Keyser U. (2017). Asymmetric dynamics of DNA entering and exiting a strongly confining nanopore. Nat. Commun..

[164] Nguyen G., Howorka S., Siwy Z.S. (2011). DNA Strands Attached Inside Single Conical Nanopores: Ionic Pore Characteristics and Insight into DNA Biophysics. J Membr. Biol..

[165] Morrin G.T., Kienle D.F., Schwartz D.K. (2021). Diffusion of Short Semiflexible DNA Polymer Chains in Strong and Moderate Confinement. ACS Macro Lett..

[166] Jonchhe S., Pandey S., Karna D., Pokhrel P., Cui Y., Mishra S., Sugiyama H., Endo M., Mao H. (2020). Duplex DNA Is Weakened in Nanoconfinement. J. Am. Chem. Soc..

[167] Jour Putnam D. (2006). Polymers for gene delivery across length scales. Nat. Mater..

[168] Dharmadi Y., Gonzalez R. (2004). DNA Microarrays: Experimental Issues, Data Analysis, and Application to Bacterial Systems. Biotechnol. Prog..

[169] Yurke B., Turberfield A.J., Mills A.P., Simmel F.C., Neumann J.L. (2000). A DNA-fuelled molecular machine made of DNA. Nature.

[170] Murphy J.C., Cano T., Fox G.E., Willson R.C. (2006). Compaction Agent Protection of Nucleic Acids during Mechanical Lysis. Biotechnol. Prog..

[171] de Lima M.C.P., Simões S., Pires P., Faneca H., Düzgüneş N. (2001). Cationic lipid–DNA complexes in gene delivery: From biophysics to biological applications. Adv. Drug Deliv. Rev..

[172] Goh S.L., Murthy N., Xu M., Fréchet J.M.J. (2004). Cross-Linked Microparticles as Carriers for the Delivery of Plasmid DNA for Vaccine Development. Bioconjugate Chem..

[173] des Rieux A., Shikanov A., Shea L.D. (2009). Fibrin hydrogels for non-viral vector delivery in vitro. J. Control. Release.

[174] Haladjova E., Rangelov S., Tsvetanov C.B., Pispas S. (2012). DNA encapsulation via nanotemplates from cationic block copolymer micelles. Soft Matter.

[175] Csaba N., Caamaño P., Sánchez A., Domínguez F., Alonso M.J. (2005). PLGA:Poloxamer and PLGA:Poloxamine Blend Nanoparticles: New Carriers for Gene Delivery. Biomacromolecules.

[176] Dimitrov I.V., Petrova E.B., Kozarova R.G., Apostolova M.D., Tsvetanov C.B. (2011). A mild and versatile approach for DNA encapsulation. Soft Matter.

[177] Park D.H., Kim J.E., Oh J.M., Shul Y.G., Choy J.H. (2010). DNA Core@Inorganic Shell. J. Am. Chem. Soc..

[178] Cruz F.J.A.L., de Pablo J.J., Mota J.P.B. (2014). Endohedral confinement of a DNA dodecamer onto pristine carbon nanotubes and the stability of the canonical B form. J. Chem. Phys..

[179] Cruz F.J., de Pablo J.J., Mota J.P. (2015). Nanoscopic Characterization of DNA within Hydrophobic Pores: Thermodynamics and Kinetics. Biochem. Eng. J..

[180] Maity A., Singh N. (2020). Melting of DNA in confined geometries. Eur. Biophys. J..

[181] Maity A., Mathur N., Imhof P., Singh N. (2022). Structural Analysis of DNA molecule in a confined shell. arXiv.

[182] Reisner W., Pedersen J.N., Austin R.H. (2012). DNA confinement in nanochannels: Physics and biological applications. Rep. Prog. Phys..

[183] Inman R.B. (1966). A denaturation map of the lambda phage DNA molecule determined by electron microscopy. J. Mol. Biol..

[184] Reisner W., Larsen N.B., Silahtaroglu A., Kristensen A., Tommerup N., Tegenfeldt J.O., Flyvbjerg H. (2010). Single-molecule denaturation mapping of DNA in nanofluidic channels. Proc. Natl. Acad. Sci. USA.

[185] Maity A., Singh A., Singh N. (2019). Stability of DNA passing through different geometrical pores. EPL Europhys. Lett..

[186] Tu B., Bai S., Lu B., Fang Q. (2018). Conic shapes have higher sensitivity than cylindrical ones in nanopore DNA sequencing. Sci. Rep..

[187] Ranton D., Deamer D.W., Marziali A., Bayley H., Benner S.A., Butler T., Di Ventra M., Garaj S., Hibbs A., Huang X. (2008). The potential and challenges of nanopore sequencing. Nat. Biotechnol..

[188] Elizabeth A.M., Derrington I.M., Laszlo A.H., Langford K.W., Hopper M.K., Gillgren N., Pavlenok M., Niederweis M., Gundlach J.H. (2012). Reading DNA at single-nucleotide resolution with a mutant MspA nanopore and phi29 DNA polymerase. Nat. Biotechnol..

[189] Kumar S., Tao C., Chien M., Hellner B., Balijepalli A., Robertson J.W., Li Z., Russo J.J., Reiner J.E., Kasianowicz J.J. (2012). PEG-Labeled Nucleotides and Nanopore Detection for Single Molecule DNA Sequencing by Synthesis. Sci. Rep..

[190] Fuller C.W., Kumar S., Porel M., Chien M., Bibillo A., Stranges P.B., Dorwart M., Tao C., Li Z., Guo W. (2016). Real-time single-molecule electronic DNA sequencing by synthesis using polymer-tagged nucleotides on a nanopore array. Proc. Natl. Acad. Sci. USA.

[191] Akeson M., Branton D., Kasianowicz J.J., Brandin E., Deamer D.W. (1999). Microsecond Time-Scale Discrimination Among Polycytidylic Acid, Polyadenylic Acid, and Polyuridylic Acid as Homopolymers or as Segments Within Single RNA Molecules. Biophys. J..

[192] Muthukumar M. (2014). Communication: Charge, diffusion, and mobility of proteins through nanopores. J. Chem. Phys..

[193] Muthukumar M. (2010). Theory of capture rate in polymer translocation. J. Chem. Phys..

[194] Rollings R., Graef E., Walsh N., Nandivada S., Benamara M., Li J. (2015). The effects of geometry and stability of solid-state nanopores on detecting single DNA molecules. Nanotechnology.

[195] (2011). Venkatesan, Bala Murali; Bashir, Rashid Nanopore sensors for nucleic acid analysis. Nat. Nanotechnol..

[196] Majd S., Erik C Y., Yazan N B., Michael X M., Jerry Y., Michael M. (2010). Applications of biological pores in nanomedicine, sensing, and nanoelectronics. Curr. Opin. Biotechnol..

[197] Schneider G.F., Kowalczyk S.W., Calado V.E., Pandraud G., Zandbergen H.W., Vandersypen L.M.K., Dekker C. (2010). DNA Translocation through Graphene Nanopores. Nano Lett..

[198] Merchant C.A., Healy K., Wanunu M., Ray V., Peterman N., Bartel J., Fischbein M.D., Venta K., Luo Z., Johnson A.T.C. (2010). DNA Translocation through Graphene Nanopores. Nano Lett..

[199] Heng J., Aksimentiev A., Ho C., Marks P., Grinkova Y., Sligar S., Schulten K., Timp G. (2006). The Electromechanics of DNA in a Synthetic Nanopore. Biophys. J..

[200] Henrickson S.E., Misakian M., Robertson B., Kasianowicz J.J. (2000). Driven DNA Transport into an Asymmetric Nanometer-Scale Pore. Phys. Rev. Lett..

[201] Henrickson S.E., DiMarzio E.A., Wang Q., Stanford V.M., Kasianowicz J.J. (2010). Probing single nanometer-scale pores with polymeric molecular rulers. J. Chem. Phys..

[202] Kasianowicz J.J., Brandin E., Branton D., Deamer D.W. (1996). Characterization of individual polynucleotide molecules using a membrane channel. Proc. Natl. Acad. Sci. USA.

[203] Perera R.T., Fleming A.M., Johnson R.P., Burrows C.J., White H.S. (2015). Detection of benzo[a]pyrene-guanine adducts in single-stranded DNA using the *α*-hemolysin nanopore. Nanotechnology.

[204] An N., White H.S., Burrows C.J. (2012). Modulation of the current signatures of DNA abasic site adducts in the [small alpha]-hemolysin ion channel. Chem. Commun..

[205] An N., Fleming A.M., White H.S., Burrows C.J. (2015). Nanopore Detection of 8-Oxoguanine in the Human Telomere Repeat Sequence. ACS Nano.

[206] Liu Z., Wang Y., Deng T., Chen Q. (2016). Solid-State Nanopore-Based DNA Sequencing Technology. J. Nanomater..

[207] Marshall M.M., Yang J., Hall A.R. (2012). Direct and Transmission Milling of Suspended Silicon Nitride Membranes With a Focused Helium Ion Beam. Scanning.

[208] Venkatesan B.M., Shah A.B., Zuo J., Bashir R. (2010). DNA Sensing Using Nanocrystalline Surface-Enhanced Al2O3 Nanopore Sensors. Adv. Funct. Mater..

[209] Postma H.W.C. (2010). Rapid Sequencing of Individual DNA Molecules in Graphene Nanogaps. Nano Lett..

[210] Saha K.K., Drndić M., Nikolić B.K. (2012). DNA Base-Specific Modulation of Microampere Transverse Edge Currents through a Metallic Graphene Nanoribbon with a Nanopore. Nano Lett..

[211] Purnell R.F., Schmidt J.J. (2009). Discrimination of Single Base Substitutions in a DNA Strand Immobilized in a Biological Nanopore. ACS Nano.

[212] Meller A., Nivon L., Brandin E., Golovchenko J., Branton D. (2000). Rapid nanopore discrimination between single polynucleotide molecules. Proc. Natl. Acad. Sci. USA.

[213] Kwok H., Briggs K., Tabard-Cossa V. (2014). Nanopore Fabrication by Controlled Dielectric Breakdown. PLoS ONE.

[214] Garaj S., Hubbard W., Reina A., Kong J., Branton D., Golovchenko J.A. (2010). Graphene as a subnanometre trans-electrode membrane. Nature.

[215] Zhang X., Price N.E., Fang X., Yang Z., Gu L.Q., Gates K.S. (2015). Characterization of Interstrand DNA–DNA Cross-Links Using the *α*-Hemolysin Protein Nanopore. ACS Nano.

[216] Song L., Hobaugh M.R., Shustak C., Cheley S., Bayley H., Gouaux J.E. (1996). Structure of Staphylococcal *α*-Hemolysin, a Heptameric Transmembrane Pore. Science.

[217] Deamer D.W., Branton D. (2002). Characterization of Nucleic Acids by Nanopore Analysis. Accounts Chem. Res..

[218] Mathé J., Visram H., Viasnoff V., Rabin Y., Meller A. (2004). Nanopore Unzipping of Individual DNA Hairpin Molecules. Biophys. J..

[219] Lin Y., Shi X., Liu S.C., Ying Y.L., Li Q., Gao R., Fathi F., Long Y.T., Tian H. (2017). Characterization of DNA duplex unzipping through a sub-2 nm solid-state nanopore. Chem. Commun..

